# Maternal Pre-conception Body Mass Index and Fasting Plasma Glucose With the Risk of Pre-term Birth: A Cohort Study Including 4.9 Million Chinese Women

**DOI:** 10.3389/frph.2021.622346

**Published:** 2021-06-15

**Authors:** Qin Xu, Qiongjie Zhou, Ying Yang, Fangchao Liu, Long Wang, Qiaomei Wang, Haiping Shen, Zongyu Xu, Yiping Zhang, Donghai Yan, Zuoqi Peng, Yuan He, Yuanyuan Wang, Ya Zhang, Hongguang Zhang, Xu Ma, Xiaotian Li

**Affiliations:** ^1^National Research Institute for Health and Family Planning, Beijing, China; ^2^Graduate School of Peking Union Medical College, Beijing, China; ^3^Obstetrics and Gynecology Hospital of Fudan University, Shanghai, China; ^4^Shanghai Key Laboratory of Female Reproductive Endocrine-Related Diseases, Shanghai, China; ^5^China DOHaD Research Center, National Human Genetic Resources Center, Beijing, China; ^6^Department of Epidemiology, National Center for Cardiovascular Diseases, Fuwai Hospital, Chinese Academy of Medical Sciences and Peking Union Medical College, Beijing, China; ^7^School of Public Health, Institute of Epidemiology and Statistics, Lanzhou University, Lanzhou, China; ^8^Department of Maternal and Child Health, National Health Commission of the People's Republic of China, Beijing, China; ^9^Institute of Biomedical Sciences, Fudan University, Shanghai, China

**Keywords:** body mass index, cohort study, fasting plasma glucose, pre-conception, pre-term birth

## Abstract

**Background:** To evaluate the associations of pre-conception body mass index (BMI), fasting plasma glucose (FPG) alone and their combination with pre-term birth (PTB) risk.

**Methods:** We conducted a population-based retrospective cohort study with 4,987,129 reproductive-aged women, who participated in National Free Pre-Pregnancy Checkups Project in 2013–2016 and had a singleton delivery before December 2017 in China. All data analyses were conducted in 2018–2021.

**Results:** A total of 339,662 (6.81%) women had pre-term deliveries. Compared with women with normal weight and normal glucose, underweight and normal weight were associated with PTB among hypoglycemia women, the adjusted odd ratios (aORs) were 1.24 (95% CI: 1.05–1.48) and 1.16 (95% CI: 1.07–1.25), respectively; underweight, overweight and obesity were associated with PTB among women with normal glucose, the aORs were 1.09 (95% CI: 1.08–1.10), 1.06 (95% CI: 1.05–1.07) and 1.08 (95% CI: 1.05–1.12), respectively; all the BMI groups were significantly associated with PTB among women with pre-diabetes or diabetes (*P* < 0.05). The dose-response relationships of BMI with PTB varied in different FPG level, with U-shaped curve in normal glucose and pre-diabetes women, J-shaped in diabetes women, L-shaped in hypoglycemia women. For FPG with PTB, the dose-response relationships were U-shaped in normal weight, overweight, and obesity women, and L-shaped in underweight women.

**Conclusion:** We found that the associations of PTB with BMI varied with levels of FPG, and associations of PTB with FPG varied with levels of BMI. There was a synergistic effect on PTB risk due to abnormal weight and glycemia besides a conventional main effect derived from either of them. Achieving desirable weight and glucose control before conception should be advised.

## Introduction

Pre-term birth (PTB), defined as a birth occurring before 37 weeks' gestational age, is the leading cause of death in children under 5 years old (1). It was estimated that over 1 in 10, or 14.84 million, babies born in 2014 worldwide were pre-term (2). PTB complications account for ~35% of the world's 2.76 million annual neonatal deaths (1), and other survivors are at increased risk of a range of short-term and long-term morbidities (3). The risk of mortality and morbidity are much higher in very PTB (<34 weeks' gestation), especially in low-income and middle-income countries (2, 4, 5). From a public health perspective for policy and planning, identifying risk factors is crucial for effective prevention and reducing PTB-associated neonatal mortality and morbidity.

With the epidemic of obesity and hyperglycemia among reproductive-aged women during the past two decades, the relationship of maternal body mass index (BMI) and blood glucose levels with adverse pregnancy outcomes has been the focus of much of the existing research (6, 7). Studies reported conflicting results about the relationship between pre-conception BMI and PTB risk. Some suggested both pre-conception underweight or obesity can increase the risk of PTB (8, 9), however, a systematic review and meta-analysis in low- and middle-income countries reported null association (10). On the other hand, although pre-gestational diabetes mellitus is reported to be associated with PTB (11, 12), seldom studies precisely focus on the adverse effects of blood glucose levels before pregnancy.

National Free Pre-Pregnancy Checkups Project (NFPCP) is a national free health service with the purpose to provide free pre-conception health examinations and follow-up of pregnancy outcomes for reproductive-aged couples who planned to get pregnant within the next 6 months. Pan et al. reported underweight was associated with increased risks of PTB based on NFPCP data of 2010–2012, but they didn't explore the expose-response relationship (13). Our previous study showed that women with pre-pregnancy hyperglycemia had a small but significantly increased risk of PTB (14). The optimal pre-conception BMI and blood glucose level for a healthy pregnancy and delivery outcome need to be further explored, which will have an important impact on pre-conception medical recommendations for avoidance of PTB. In addition, both BMI and blood glucose level can reflect the body's metabolic status, obesity, and hyperglycemia have the common metabolic disorder characteristics such as increased insulin resistance and hyperinsulinemia, but seldom study has explored their combined effects on PTB. Therefore, we conducted a retrospective cohort study in China to comprehensively examine the associations of maternal pre-conception BMI and blood fasting plasma glucose (FPG) alone and their combination with PTB.

## Methods

### Study Design and Participants

We did a large population-based retrospective cohort study among 20–49 years women who participated in the NFPCP from 2013 to 2016, successfully became pregnant and subsequently delivered a singleton baby before December 2017. The project began with serving only rural married couples from 2010 to 2012, and after 2013, was further extended to both rural and urban couples across 31 provinces in mainland China. The service provided pre-conception care including free health examinations, risk assessments, consultations, early pregnancy follow-up and pregnancy outcome follow-up. All health survey data were promptly uploaded to and stored in the NFPCP medical service information system. Detailed design, organization, and implementation of this project were described elsewhere (15, 16). The study was approved by the Institutional Research Review Board at the National Research Institute for Health and Family Planning. Written informed consent was obtained from all NFPCP participants.

**Figure 1:**
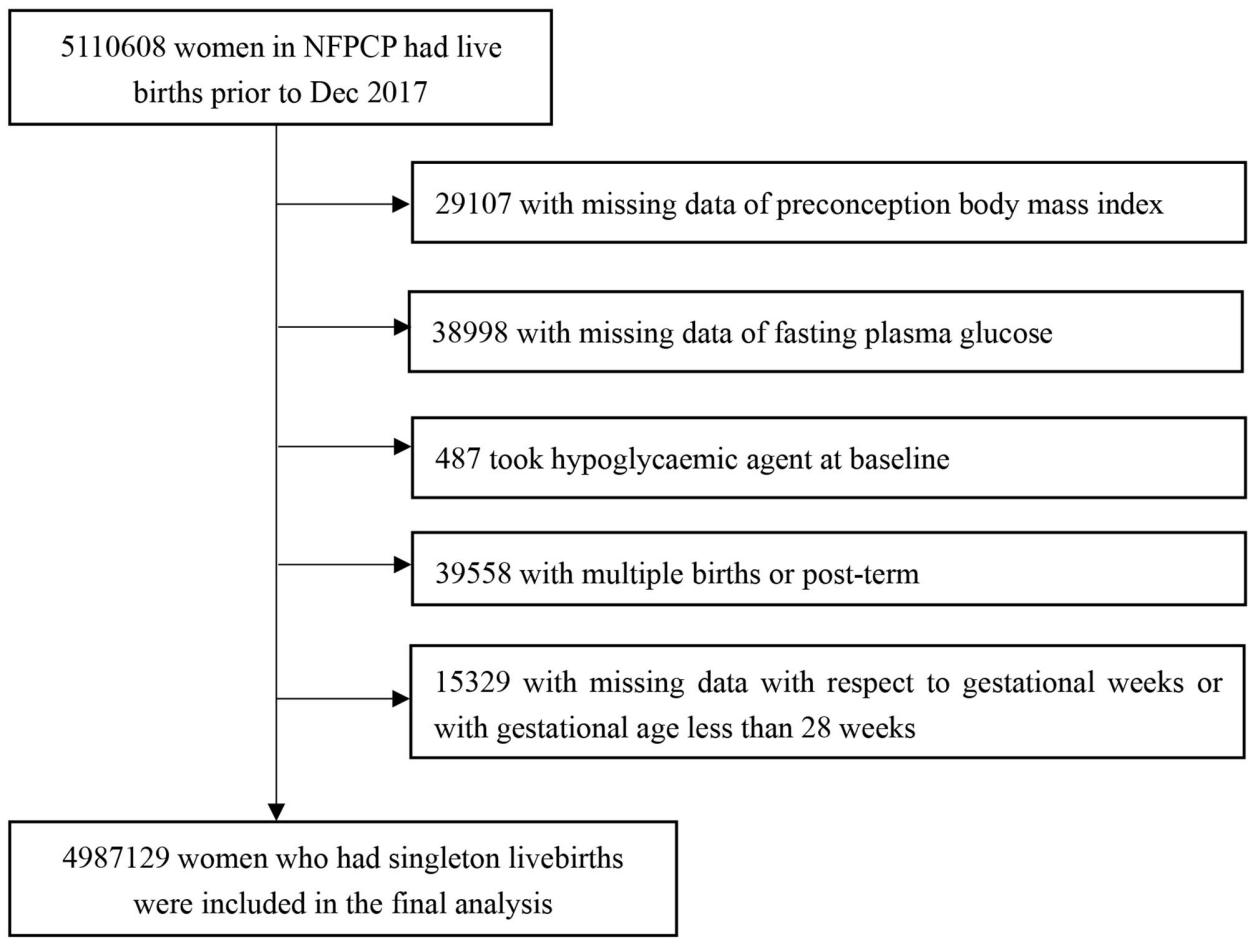
Flowchart of the study participants. NFPCP, National Free Pre-Pregnancy Checkups Project.

Women aged 20–49 years old participated in the NFPCP from January 1, 2013, to December 31, 2016, successfully got pregnant and gave live births until December 31, 2017 in China were included in the current study. The exclusion criteria were as follows: (1) women with missing data of pre-conception BMI; (2) women with missing data of FPG; (3) women who took hypoglycaemic agent at baseline; (4) women who gave multiple births or with post-term; (5) women with missing data with respect to gestational weeks or with gestational age <28 weeks. Detailed information on the study population recruitment, and derivation of the population used in the final analysis, is shown in Figure 1.

### Procedures

Information, including demographic characteristics, lifestyle, disease history, medication history, reproductive history, and other relevant factors was collected by trained staff using standard and structured questionnaire. Body weight and height were measured with participants wearing light, indoor clothes, and no shoes. BMI was calculated using weight/height^2^ (kg/m^2^), then were categorized using the WHO definition (17): underweight (<18.5 kg/m^2^), normal weight (18.5–24.9 kg/m^2^), overweight (25.0–29.9 kg/m^2^) and obesity (≥30.0 kg/m^2^). Seated blood pressure (BP) was measured using an automated BP monitor on a single occasion after participants rested ≥10 min. Hypertension was defined as systolic BP ≥ 140 mmHg or diastolic BP ≥ 90 mmHg or self-reported hypertension. Blood samples after at least 8 h fasting were taken and immediately stored at 4–8°C, then sent to the local laboratories for analysis within 24 h. FPG was measured using glucose oxidase or hexokinase methods in the local laboratories in accordance with National Guide to Clinical Laboratory Procedures. FPG categories was defined according to the American Diabetes Association (18): hypoglycemia ( ≤ 2.7 mmol/L), normal glucose level (2.8–5.5 mmol/L), pre-diabetes (5.6–6.9 mmol/L), and diabetes (≥7.0 mmol/L). Thyroid dysfunction was defined as thyroid-stimulating hormone <0.44 or >3.45 mIU/ml, or a history of thyroid disease (19). Anemia was defined as hemoglobin concentration <110 g/L. Infectious disease was defined as hepatitis B surface antigen positive, or *Treponema pallidum, Neisseria gonorrhoeae*, or *Chlamydia trachomatis* positive during pre-conception physical examination.

Trained local health staff were assigned to interview participants by telephone to track their pregnancy status within 3 months after pre-conception examination. If the participants did not get pregnant at the first interview, repeated surveys were conducted within the next 3 months until 1 year after baseline examination. Information about the last menstrual period (LMP), smoking, and alcohol consumption status during early pregnancy was collected. Women who had become pregnant were contacted again to ascertain their pregnancy outcomes within 1 year after the early pregnancy follow-up completed. Self-reported delivery date, delivery mode, and neonate conditions were collected.

### Outcomes

The primary outcome was PTB, defined as births delivered at gestational ages between 28 and 36 + 6 weeks. Then moderate PTB (MPTB, 34 to <37 weeks) and very PTB (VPTB, 28 to <34 weeks) were further defined. In the current study, gestational weeks was the calculated according to the date of delivery and the first day of the LMP. During early pregnancy, women reported pregnant were asked to come back to the clinic to undergo ultrasonic examinations and have a physician's diagnosis to confirm the pregnancy about 2 months after the LMP, and the first day of the LMP was adjusted by ultrasonic examinations this time.

### Statistical Analysis

Baseline characteristics were presented as number (percentage) for categorical variables according to BMI and FPG groups, respectively. The χ^2^ test or Kruskal-Wallis test was used to compare the distributions of baseline characteristics according to different BMI or FPG groups.

Firstly, we constructed a 16-level variable that combined 4 levels of BMI with 4 levels of FPG to examine the combined effects of BMI and FPG on PTB, as well as MPTB and VPTB. The crude and multivariate-adjusted logistic regression models were separately used to estimate the ORs and 95% confidence intervals (CIs) using normal weight and normal glucose as the reference group. Then, we assessed the dose-response relationships of BMI with the risk of PTB stratified by FPG categories, as well as with the risk of MPTB and VPTB, using restricted cubic spline (RCS) models. The dose-response relationships of FPG with outcomes stratified by BMI categories were also explored. We plotted smooth curves with four knots at the 5th, 35th, 65th, and 95th percentiles of BMI or FPG. We chose 4 knots because it yielded better model fit compared to using 3 or 5 knots assessed by Akaike information criteria. The non-linearity of the dose response was tested by Wald statistics (20).

Covariates in the multivariate-adjusted models included age at LMP (20–24, 25–29, 30–34, 35–39, or 40–49 years), level of education (senior high school or higher, junior high school or below), ethnicity (Han, others), occupation (farmers, others), smoking before or during early pregnancy (yes, no), and alcohol drinking before or during early pregnancy (yes, no), parity (nullipara: no previous live birth, multipara: 1 or more live births), history of adverse pregnancy outcomes, including history of spontaneous abortion, stillbirth, pre-term birth, or induced abortion (yes, no), hypertension (yes, no), thyroid dysfunction (yes, no), anemia (yes, no), infectious disease (yes, no), and sex of child (male, female). All missing value of categorical variables was recoded as new category in the models. All the RCS models were adjusted for covariates mentioned above.

To examine the robustness of our findings, we also conducted sensitivity analyses by using Chinese criterion of BMI categories: <18.5, 18.5–23.9, 24.0–27.9, and ≥28.0 kg/m^2^. Sensitivity analyses were also conducted by excluding participants with self-reported diabetes and other chronic diseases (including heart disease, epilepsy, chronic nephritis, tumor, and mental disorders.), and according to delivery type (cesarean or not). All analyses were performed using R, version 3.2.2 (Development Core Team, 2015). *P* <0.05 were considered statistically significant (two-sided). All data analyses were conducted in 2018–2021.

## Results

By December 31, 2017, 5,110,608 women had live births, we then further excluded 29,107 and 38,998 women with missing data of pre-conception BMI or FPG, 487 women who took hypoglycaemic agent at baseline, 39,558 women with multiple births or post-term, 15,329 women with missing data on gestational weeks or with gestational age <28 weeks. The remaining 4,987,129 women were included in the final analysis (Figure 1). The baseline comparison between included and excluded participants is given in Supplementary Table 1.

The median age of the participants was 25.0 years (interquartile range: 23.0–28.0 years). Overall, 13.5% (675,297) women were underweight, and 9.7% (479,431) were overweight (8.4%, 416,706) or obesity (1.3%, 626,86) (Table 1). Proportion of hyperglycemia was 13.7% (686,725), with 12.8% of pre-diabetes (639,661) and 0.9% of diabetes (47,064), respectively. The baseline characteristics of participants, according to maternal pre-conception BMI or FPG, showed that women who were overweight, obesity, pre-diabetes, or diabetes were more likely to be older, have a history of adverse pregnancy outcomes, hypertension and be cesarean.

**Table 1:** Demographic and clinical characteristics by pre-conception body mass index and fasting plasma glucose.

	**Total participants (*n* = 4,987,129)**	Pre-term birth* (*n* = 339,662)	**BMI (kg/m** ^**2**^ )^*^				FPG (mmol/L)^*^			
			**Underweight (*n* = 675,297)**	**Normal weight (*n* = 3,832,440)**	**Overweight (*n* = 416,706)**	**Obesity (*n* = 62,686)**	**Hypoglycemia (*n* = 11,088)**	**Normal glucose (*n* = 4,289,316)**	**Pre-diabetes (*n* =639,661)**	**Diabetes (*n* = 47064)**
**Age at last menstrual, years**										
20–24	1,994,885 (40.0)	137,873 (6.9)	325,479 (48.2)	1,529,893 (39.9)	121,208 (29.1)	18,305 (29.2)	4,905 (44.2)	1,738,734 (40.5)	235,289 (36.8)	15,957 (33.9)
25–29	2,115,194 (42.4)	138,975 (6.6)	289,089 (42.8)	1,624,594 (42.4)	174,330 (41.8)	27,181 (43.4)	4,498 (40.6)	1,822,564 (42.5)	268,691 (42.0)	19,441 (41.3)
30–34	642,345 (12.9)	45,747 (7.1)	50,269 (7.4)	498,119 (13.0)	81,857 (19.6)	12,100 (19.3)	1,222 (11.0)	538,206 (12.5)	95,191 (14.9)	7,726 (16.4)
35–39	199,390 (4.0)	14,407 (7.2)	9,377 (1.4)	153,253 (4.0)	32,475 (7.8)	4,285 (6.8)	381 (3.4)	162,113 (3.8)	3,3741 (5.3)	3,155 (6.7)
40–49	35,315 (0.7)	2,660 (7.5)	1,083 (0.2)	26,581 (0.7)	6,836 (1.6)	815 (1.3)	82 (0.7)	27,699 (0.6)	6,749 (1.1)	785 (1.7)
**Ethnicity**										
Han	4,591,716 (92.1)	308,094 (6.7)	620,734 (91.9)	3,525,408 (92.0)	386,870 (92.8)	58,704 (93.6)	9,359 (84.4)	3,947,872 (92.0)	592,078 (92.6)	42,407 (90.1)
Others	338,755 (6.8)	27,877 (8.2)	45,184 (6.7)	263,955 (6.9)	26,127 (6.3)	3,489 (5.6)	1,660 (15.0)	292,707 (6.8)	40,509 (6.3)	3,879 (8.2)
NA	56,658 (1.1)	3,691 (6.5)	9,379 (1.4)	43,077 (1.1)	3,709 (0.9)	493 (0.8)	69 (0.6)	4,8737 (1.1)	7,074 (1.1)	778 (1.7)
**Education**										
Senior high school or higher	1,808,064 (36.3)	112,024 (6.2)	305,023 (45.2)	1,371,482 (35.8)	116,310 (27.9)	15,249 (24.3)	4,086 (36.9)	1,567,444 (36.5)	220,391 (34.5)	16,143 (34.3)
Junior high school or below	3,035,338 (60.9)	217,764 (7.2)	347,015 (51.4)	2,352,185 (61.4)	290,175 (69.6)	45,963 (73.3)	6,818 (61.5)	2,596,024 (60.5)	403,091 (63.0)	29,405 (62.5)
NA	143727 (2.9)	9,874 (6.9)	23,259 (3.4)	108,773 (2.8)	10,221 (2.5)	1,474 (2.4)	184 (1.7)	125,848 (2.9)	16,179 (2.5)	1,516 (3.2)
**Occupation**										
Farmers	3,537,200 (70.9)	247,659 (7.0)	417,203 (61.8)	2,747,490 (71.7)	322,632 (77.4)	49,875 (79.6)	7,638 (68.9)	3,039,556 (70.9)	458,112 (71.6)	31,894 (67.8)
Others	1,293,287 (25.9)	81,161 (6.3)	231,444 (34.3)	966,994 (25.2)	83,594 (20.1)	11,255 (18.0)	3,204 (28.9)	1,113,928 (26.0)	162,750 (25.4)	13,405 (28.5)
NA	156,642 (3.1)	108,42 (6.9)	26,650 (3.9)	117,956 (3.1)	10,480 (2.5)	1,556 (2.5)	246 (2.2)	135,832 (3.2)	18,799 (2.9)	17,65 (3.8)
**Parity**										
Nullipara	3,043,053 (61.0)	189,094 (6.2)	500,658 (74.1)	2343,080 (61.1)	173,563 (41.7)	25,752 (41.1)	7,252 (65.4)	264,7371 (61.7)	361,356 (56.5)	27,074 (57.5)
Multipara	1,929,055 (38.7)	149,681 (7.8)	172,077 (25.5)	1478,033 (38.6)	242,163 (58.1)	36,782 (58.7)	3,819 (34.4)	1,629,273 (38.0)	276,189 (43.2)	19,774 (42.0)
NA	15,021 (0.3)	887 (5.9)	2,562 (0.4)	11,327 (0.3)	980 (0.2)	152 (0.2)	17 (0.2)	12,672 (0.3)	2,116 (0.3)	216 (0.5)
**Smoking**										
No	4,899,644 (98.2)	331,470 (6.8)	661,948 (98)	3,766,886 (98.3)	409,338 (98.2)	61,472 (98.1)	10,820 (97.6)	4,214,530 (98.3)	628,231 (98.2)	46,063 (97.9)
Yes	24,801 (0.5)	2,128 (8.6)	3,750 (0.6)	18,165 (0.5)	2,415 (0.6)	471 (0.8)	58 (0.5)	21,271 (0.5)	3,206 (0.5)	266 (0.6)
NA	62,684 (1.3)	6,064 (9.7)	9,599 (1.4)	47,389 (1.2)	4,953 (1.2)	743 (1.2)	210 (1.9)	53,515 (1.2)	8,224 (1.3)	735 (1.6)
**Alcohol drinking**										
No	4,767,062 (95.6)	322,666 (6.8)	638,270 (94.5)	3,668,230 (95.7)	400,341 (96.1)	60,221 (96.1)	10,387 (93.7)	4,099,874 (95.6)	612,025 (95.7)	44,776 (95.1)
Yes	151,280 (3.0)	10,501 (6.9)	26,557 (3.9)	112,143 (2.9)	10,939 (2.6)	1,641 (2.6)	479 (4.3)	130,748 (3.0)	18,597 (2.9)	1,456 (3.1)
NA	68,787 (1.4)	6,495 (9.4)	10,470 (1.6)	52,067 (1.4)	5,426 (1.3)	824 (1.3)	222 (2.0)	58,694 (1.4)	9,039 (1.4)	832 (1.8)
**Adverse pregnancy history** ^**a**^										
No	4,157,294 (83.4)	281,205 (6.8)	564,728 (83.6)	3,221,099 (84.0)	323,346 (77.6)	48,121 (76.8)	9,562 (86.2)	3,586,415 (83.6)	523,475 (81.8)	37842 (80.4)
Yes	814,810 (16.3)	57,570 (7.1)	108,006 (16.0)	600,012 (15.7)	92,379 (22.2)	14,413 (23.0)	1,508 (13.6)	690,225 (16.1)	11,4071 (17.8)	9,006 (19.1)
NA	15,025 (0.3)	887 (5.9)	2,563 (0.4)	11,329 (0.3)	981 (0.2)	152 (0.2)	18 (0.2)	12,676 (0.3)	2,115 (0.3)	216 (0.5)
**Hypertension**										
No	4,905,329 (98.4)	332,349 (6.8)	667,573 (98.9)	3,779,356 (98.6)	400,547 (96.1)	57,853 (92.3)	10,918 (98.5)	4,223,958 (98.5)	624,913 (97.7)	45,540 (96.8)
Yes	74,039 (1.5)	6,807 (9.2)	6,497 (1.0)	47,269 (1.2)	15,526 (3.7)	4,747 (7.6)	157 (1.4)	58,669 (1.4)	13,758 (2.2)	1,455 (3.1)
NA	7,761 (0.2)	506 (6.5)	1,227 (0.2)	5,815 (0.2)	633 (0.2)	86 (0.1)	13 (0.1)	6,689 (0.2)	990 (0.2)	69 (0.1)
**Thyroid dysfunction**										
No	4,403,374 (88.3)	296,556 (6.7)	591,602 (87.6)	3,393,981 (88.6)	363,543 (87.2)	54,248 (86.5)	9,597 (86.6)	3,793,092 (88.4)	560,531 (87.6)	40,154 (85.3)
Yes	576,630 (11.6)	42,639 (7.4)	82,589 (12.2)	433,109 (11.3)	52,575 (12.6)	8,357 (13.3)	1,478 (13.3)	490,102 (11.4)	78,204 (12.2)	6,846 (14.5)
NA	7,125 (0.1)	467 (6.6)	1,106 (0.2)	5,350 (0.1)	588 (0.1)	81 (0.1)	13 (0.1)	6,122 (0.1)	926 (0.1)	64 (0.1)
**Anemia**										
No	4,646,641 (93.2)	317,323 (6.8)	625,505 (92.6)	3,569,736 (93.1)	391,997 (94.1)	59,403 (94.8)	10,064 (90.8)	3,998,150 (93.2)	595,609 (93.1)	42,818 (91.0)
Yes	328,191 (6.6)	21,335 (6.5)	48,063 (7.1)	253,180 (6.6)	23,804 (5.7)	3,144 (5.0)	958 (8.6)	280,731 (6.5)	42,416 (6.6)	4,086 (8.7)
NA	12,297 (0.2)	1,004 (8.2)	1,729 (0.3)	9,524 (0.2)	905 (0.2)	139 (0.2)	66 (0.6)	10,435 (0.2)	1,636 (0.3)	160 (0.3)
**Infectious disease**										
No	4,502,465 (90.3)	303,260 (6.7)	592,599 (87.8)	3,470,953 (90.6)	381,508 (91.6)	57,405 (91.6)	9,391 (84.7)	3,879,220 (90.4)	573,687 (89.7)	40,167 (85.3)
Yes	265,087 (5.3)	18,976 (7.2)	42,155 (6.2)	200,292 (5.2)	19,673 (4.7)	2,967 (4.7)	805 (7.3)	225,779 (5.3)	35,001 (5.5)	3,502 (7.4)
NA	219,577 (4.4)	17,426 (7.9)	40,543 (6)	161,195 (4.2)	15,525 (3.7)	2,314 (3.7)	892 (8.0)	184,317 (4.3)	30,973 (4.8)	3,395 (7.2)
**Neonatal sex**										
Male	2,591,998 (52.0)	184,522 (7.1)	350,268 (51.9)	1,992,721 (52.0)	216,541 (52.0)	32,468 (51.8)	5,729 (51.7)	2,228,874 (52.0)	332,869 (52.0)	24,526 (52.1)
Female	2,389,131 (47.9)	152,657 (6.4)	324,260 (48.0)	1,835,225 (47.9)	199,525 (47.9)	30,121 (48.1)	5,350 (48.3)	2,055,300 (47.9)	306,027 (47.8)	22,454 (47.7)
NA	6,000 (0.1)	2,483 (41.4)	769 (0.1)	4,494 (0.1)	640 (0.2)	97 (0.2)	9 (0.1)	5,142 (0.1)	765 (0.1)	84 (0.2)
**Cesarean**										
No	3,553,810 (71.3)	235,895 (6.6)	508,857 (75.4)	2,756,104 (71.9)	253,742 (60.9)	35,107 (56.0)	8,267 (74.6)	3,067,141 (71.5)	446,046 (69.7)	32,356 (68.7)
Yes	1,433,319 (28.7)	103,767 (7.2)	166,440 (24.6)	1,076,336 (28.1)	162,964 (39.1)	27,579 (44.0)	2,821 (25.4)	1,222,175 (28.5)	193,615 (30.3)	14,708 (31.3)
**Habitant**										
Rural	4,549,991 (91.2)	310,398 (6.8)	605,056 (89.6)	3,502,095 (91.4)	384,336 (92.2)	58,504 (93.3)	9,954 (89.8)	3,910,579 (91.2)	586,319 (91.7)	43,139 (91.7)
Urban	43,6711 (8.8)	29,236 (6.7)	70,163 (10.4)	330,042 (8.6)	32,335 (7.8)	4,171 (6.7)	1,134 (10.2)	378,339 (8.8)	53,317 (8.3)	3,921 (8.3)
NA	427 (0)	28 (6.6)	78 (0)	303 (0)	35 (0)	11 (0)	0 (0)	398 (0)	25 (0)	4 (0)

^a^*Adverse pregnancy history included history of spontaneous abortion, stillbirth, pre-term birth, or induced abortion*.

* *The group differences between pre-term birth and term birth, BMI groups or FPG groups, with respect for the baseline characteristics were all statistically significant (p <0.05), except for neonatal sex in BMI groups or FPG groups and smoking in FPG groups (p > 0.05)*.

*BMI, body mass index; FPG, fasting plasma glucose. Underweight (BMI <18.5 kg/m^2^); normal weight (BMI 18.5–24.9 kg/m^2^); overweight (BMI 25.0–29.9 kg/m^2^); obesity (BMI ≥ 30.0 kg/m^2^); hypoglycemia (FPG ≤ 2.7 mmol/L); normal glucoses (FPG 2.8–5.5 mmol/L); pre-diabetes (FPG 5.6–6.9 mmol/L); diabetes (FPG ≥ 7.0 mmol/L)*.

The median length of time from baseline examination to pregnancy was 1.13 months (IQR: 0.39–3.53). A total of 339,662 PTB events were documented in the 4,987,129 women, and the overall cumulative incidence of PTB was 6.81% (95% CI: 6.79–6.83%). In general, as pre-conception FPG increased, the amount of overweight and obese women increased (Table 2). For example, 7.3% of hypoglycemia women were overweight and obesity, whereas, 19.0% of diabetes women were overweight and obesity.

**Table 2:** The combined associations of maternal pre-conception BMI and FPG with PTB, MPTB, and VPTB.

**Classification**		**No. of participants**	**PTB**			**Moderate pre-term birth**			**Very pre-term birth**		
			***n* (%)**	**Crude OR (95% CI)**	**Multivariable adjusted OR (95% CI)^**#**^**	***n* (%)**	**Crude OR (95% CI)**	**Multivariable adjusted OR (95% CI)^**#**^**	***n* (%)**	**Crude OR (95% CI)**	**Multivariable adjusted OR (95% CI)^**#**^**
Hypoglycemia	Underweight	1,752 (15.8)	140 (8.0)	1.22 (1.03–1.45)	1.24 (1.05–1.48)	95 (5.6)	1.17 (0.95–1.44)	1.18 (0.96–1.46)	45 (2.7)	1.35 (1.00–1.82)	1.4 (1.04–1.88)
	Normal weight	8,530 (76.9)	663 (7.8)	1.19 (1.10–1.29)	1.16 (1.07–1.25)	455 (5.5)	1.15 (1.05–1.26)	1.13 (1.03–1.24)	208 (2.6)	1.28 (1.11–1.47)	1.23 (1.07–1.42)
	Overweight	721 (6.5)	44 (6.1)	0.92 (0.68–1.24)	0.84 (0.62–1.14)	32 (4.5)	0.94 (0.66–1.34)	0.87 (0.61–1.24)	12 (1.7)	0.86 (0.48–1.52)	0.77 (0.44–1.36)
	Obesity	85 (0.8)	10 (11.8)	1.88 (0.97–3.63)	1.76 (0.91–3.41)	5 (6.3)	1.33 (0.54–3.28)	1.25 (0.50–3.09)	5 (6.3)	3.22 (1.30–7.97)	2.98 (1.20–7.38)
Normal glucose	Underweight	590,603 (13.8)	41,374 (7.0)	1.06 (1.05–1.07)	1.09 (1.08–1.10)	29,862 (5.2)	1.08 (1.07–1.10)	1.11 (1.09–1.12)	11,512 (2.1)	1.01 (0.99–1.03)	1.06 (1.04–1.08)
	Normal weight	3,311,276 (77.2)	219,480 (6.6)	1	1	155,535 (4.8)	1	1	63,945 (2.0)	1	1
	Overweight	338,721 (7.9)	24,840 (7.3)	1.12 (1.10–1.13)	1.06 (1.05–1.07)	17,831 (5.4)	1.13 (1.11–1.15)	1.08 (1.06–1.10)	7,009 (2.2)	1.08 (1.05–1.11)	1.01 (0.99–1.04)
	Obesity	48,716 (1.1)	3,699 (7.6)	1.16 (1.12–1.20)	1.08 (1.05–1.12)	2,586 (5.4)	1.14 (1.10–1.19)	1.08 (1.03–1.12)	1,113 (2.4)	1.20 (1.13–1.27)	1.10 (1.04–1.17)
Pre-diabetes	Underweight	77,022 (12.0)	5,444 (7.1)	1.07 (1.04–1.10)	1.09 (1.06–1.12)	3,899 (5.2)	1.08 (1.05–1.12)	1.10 (1.06–1.14)	1,545 (2.1)	1.04 (0.99–1.10)	1.08 (1.03–1.14)
	Normal weight	480,448 (75.1)	33,345 (6.9)	1.05 (1.04–1.06)	1.04 (1.03–1.05)	23,896 (5.1)	1.06 (1.05–1.08)	1.05 (1.04–1.07)	9,449 (2.1)	1.02 (1.00–1.04)	1.01 (0.99–1.03)
	Overweight	70,218 (11.0)	5,804 (8.3)	1.27 (1.24–1.30)	1.19 (1.16–1.22)	4,128 (6.0)	1.27 (1.23–1.32)	1.20 (1.16–1.24)	1,676 (2.5)	1.26 (1.20–1.32)	1.18 (1.12–1.24)
	Obesity	11,973 (1.9)	1,027 (8.6)	1.32 (1.24–1.41)	1.21 (1.14–1.29)	736 (6.3)	1.34 (1.24–1.44)	1.24 (1.15–1.33)	291 (2.6)	1.29 (1.14–1.44)	1.16 (1.03–1.31)
Diabetes	Underweight	5,920 (12.6)	447 (7.6)	1.15 (1.05–1.27)	1.18 (1.07–1.30)	313 (5.4)	1.14 (1.01–1.27)	1.15 (1.03–1.29)	134 (2.4)	1.18 (1.00–1.41)	1.23 (1.04–1.46)
	Normal weight	32,186 (68.4)	2,495 (7.8)	1.18 (1.14–1.23)	1.16 (1.12–1.21)	1,749 (5.6)	1.17 (1.12–1.23)	1.15 (1.10–1.21)	746 (2.5)	1.22 (1.13–1.31)	1.20 (1.11–1.29)
	Overweight	7,046 (15.0)	645 (9.2)	1.42 (1.31–1.54)	1.32 (1.21–1.43)	469 (6.8)	1.46 (1.33–1.60)	1.36 (1.23–1.49)	176 (2.7)	1.33 (1.14–1.55)	1.23 (1.05–1.43)
	Obesity	1,912 (4.0)	205 (10.7)	1.69 (1.46–1.96)	1.53 (1.33–1.78)	148 (8.0)	1.72 (1.46–2.04)	1.56 (1.32–1.85)	57 (3.2)	1.62 (1.24–2.10)	1.48 (1.13–1.93)

*BMI, body mass index; FPG, fasting plasma glucose; PTB, pre-term birth (28 to <37 weeks); MPTB, moderate pre-term birth (34 to <37 weeks); VPTB, very pre-term birth (28 to <34 weeks); OR, odd ratio. Multivariable-adjusted OR (95% CI) were adjusted for characteristics of women (age, education, ethnic, occupation), parity, smoking and alcohol drinking before or during early pregnancy, history of adverse pregnancy outcomes, history of hypertension, thyroid dysfunction, anemia, infectious disease, and sex of child*.

Compared with women in the reference group, underweight and normal weight were associated with PTB among hypoglycemia women, the multivariate-adjusted ORs were 1.24 (95% CI: 1.05–1.48) and 1.16 (95% CI: 1.07–1.25), respectively; the ORs were 1.09 (95% CI: 1.08–1.10), 1.06 (95% CI: 1.05–1.07) and 1.08 (95% CI: 1.05–1.12) for underweight, overweight, and obesity among women with normal glucose, respectively; all the pre-conception BMI groups were associated with PTB among women with pre-diabetes or diabetes. The ORs were 1.09 (95% CI: 1.06–1.12), 1.04 (95% CI: 1.03–1.05), 1.19 (95% CI: 1.16–1.22) and 1.21 (95% CI: 1.14–1.29) for underweight, normal weight, overweight, and obesity among women with pre-diabetes, respectively; the corresponding ORs were 1.18 (95% CI: 1.07–1.30), 1.16 (95% CI: 1.12–1.21), 1.32 (95% CI: 1.21–1.43) and 1.53 (95% CI: 1.33–1.78) among women with diabetes. Similar results were observed for MPTB and VPTB (Table 2; Supplementary Figure 5). In the sensitivity analyses, the associations between pre-conception BMI, FPG and risk of PTB, as well as MPTB and VPTB, did not change appreciably by using Chinese criterion of BMI categories (Supplementary Table 2), or after excluding participants with self-reported diabetes (*n* = 549) and other chronic diseases (*n* = 8,259) (Supplementary Table 3), or in cesarean group (Supplementary Table 4).

**Figure 2:**
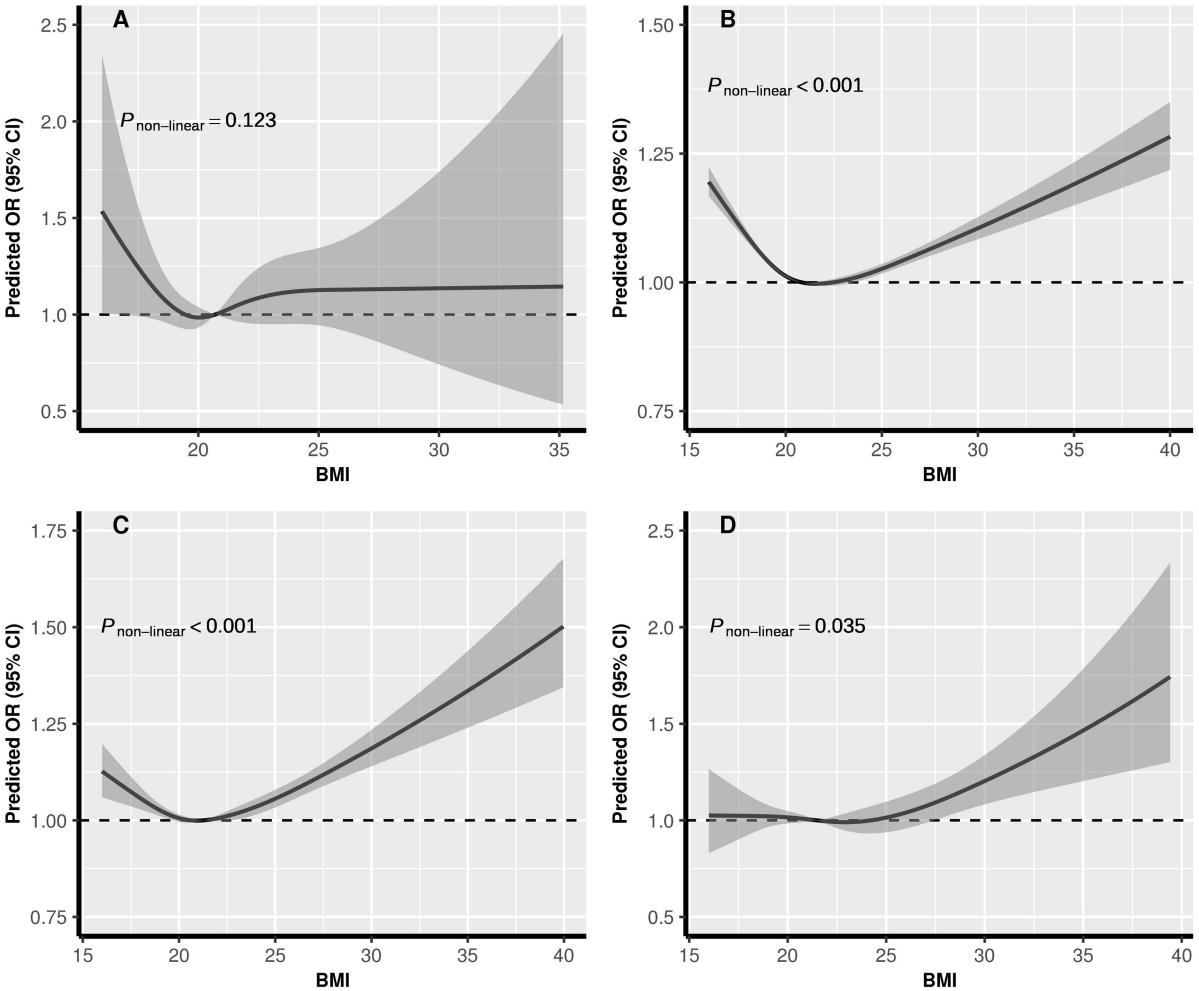
The exposure-response relationship of pre-conception BMI with PTB, stratified by FPG. BMI, body mass index; FPG, fasting plasma glucose; PTB, (pre-term birth, 28 to <37 weeks). **(A)** Hypoglycemia; **(B)** Normal glucose; **(C)** Pre-diabetes; **(D)** Diabetes. In the graph, black lines, and shaded gray areas represent predicted ORs and 95% CIs, respectively. The models were adjusted for characteristics of women (age, education, ethnic, occupation), parity, smoking and alcohol drinking before or during early pregnancy, history of adverse pregnancy outcomes, history of hypertension, thyroid dysfunction, anemia, infectious disease, and sex of child.

**Figure 3:**
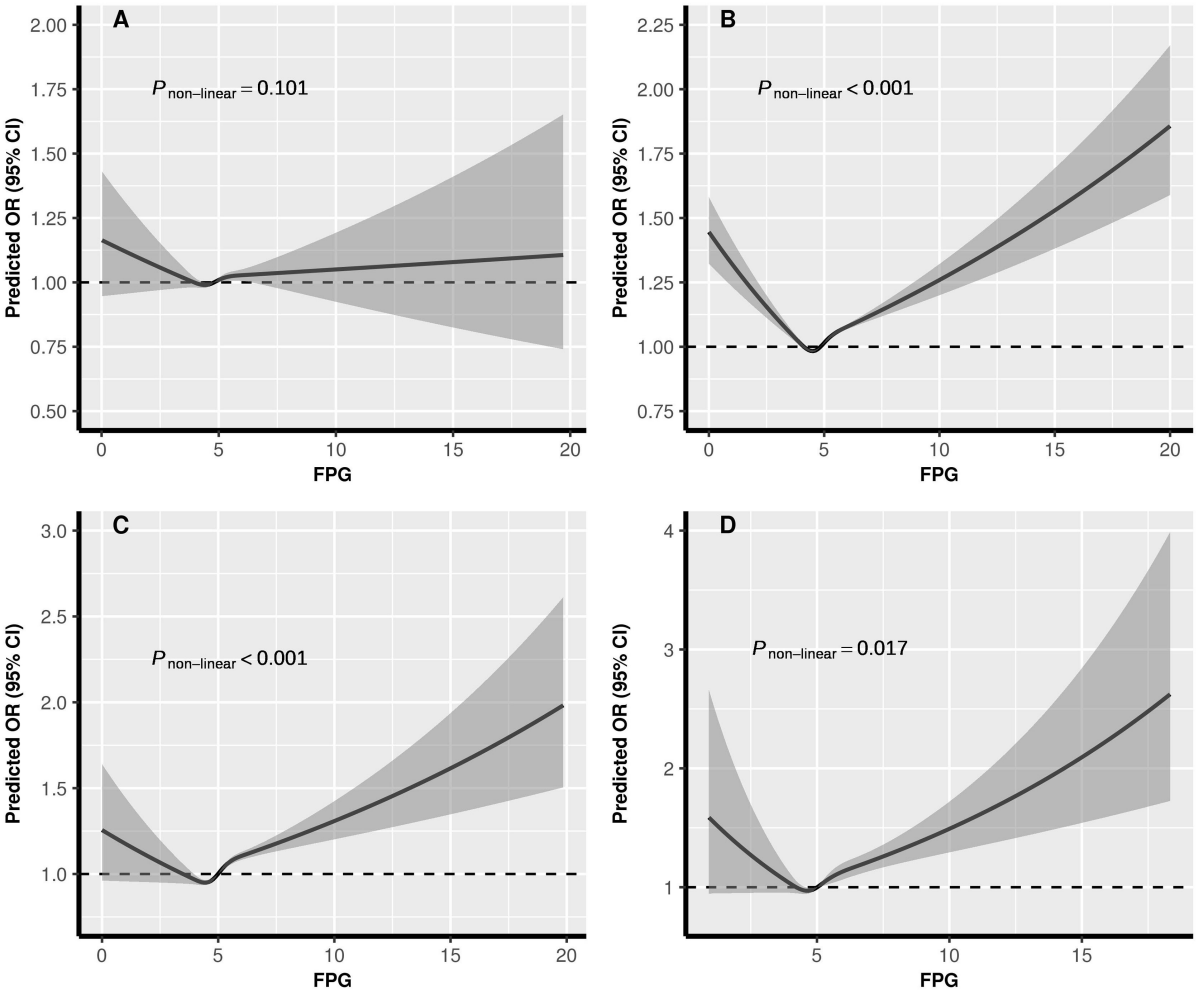
The exposure-response relationship of pre-conception FPG with PTB, stratified by BMI. BMI, body mass index; FPG, fasting plasma glucose; PTB, (pre-term birth, 28 to <37 weeks). **(A)** Underweight; **(B)** Normal weight; **(C)** Overweight; **(D)** Obesity. In the graph, black lines and shaded gray areas represent predicted ORs and 95% CIs, respectively. The models were adjusted for characteristics of women (age, education, ethnic, occupation), parity, smoking and alcohol drinking before or during early pregnancy, history of adverse pregnancy outcomes, history of hypertension, thyroid dysfunction, anemia, infectious disease, and sex of child.

The dose-response relationships between pre-conception BMI and the risk of PTB, stratified by FPG, were depicted in Figure 2. The shapes of pre-conception BMI and the risk of PTB were generally U-shaped in normal glucose and pre-diabetes women (*P*_*non*−*linear*_ <0.05), and J-shaped in diabetes women, the risk of PTB increased with the increase of BMI (*P*_*non*−*linear*_ = 0.035). The curve in hypoglycemia women was approximately L-shaped (*P*_*non*−*linear*_ = 0.123). As shown in Figure 3, U-shaped relationships of pre-conception FPG and the risk of PTB in normal weight, overweight, and obesity women were identified (*P*_*non*−*linear*_ <0.05). The curve in underweight women was approximately L-shaped, with increased risk of PTB in the lower FPG level (*P*_*non*−*linear*_ = 0.101). The dose-response relationships between pre-conception BMI and the risk of MPTB and VPTB, stratified by FPG were shown in Supplementary Figures 2, 3. The dose-response relationships between pre-conception FPG and the risk of MPTB and VPTB, stratified by BMI were shown in Supplementary Figures 4, 5.

## Discussion

In this large-scale population-based retrospective cohort study including over 4.9 million Chinese women, we identified that the associations between PTB and BMI varied with levels of FPG, and the highest estimated risk of PTB was identified in obese women with diabetes. In addition, the associations were consistent in categorical, dose–response manners, and in subgroup of PTB, including MPTB and VPTB. Our findings provided supportive evidence for achieving desirable weight and glucose control before conception in purpose to eliminate potential risk of pre-term birth.

The association of maternal pre-conception BMI and PTB has been widely explored in previous studies, most of which consistently reported pre-conception underweight was a risk factor of PTB (21, 22), while the findings for overweight or obesity were conflicting (10, 23). These inconsistent results can be partly attributed to the different definitions of PTB. The systematic review conducted by Torloni MR et al. showed a deleterious effect of maternal obesity on overall PTB, but suggested a protective effect against spontaneous PTB (23). Our study identified a U-shaped relationship between pre-conception BMI and PTB risk in normal glucose and pre-diabetes women using BMI as a continuous variable in the RCS model, revealing that underweight or overweight/obese women with normal glucose or pre-diabetes were both at higher risk of PTB. This is consistent with previous studies (8, 9) and another cohort study including 536,098 women of NFPCP in rural China (13). Different from us, they all divided participants into underweight, normal weight, overweight, or obesity based on conventional BMI cut-offs (8, 9, 13).

The potential mechanisms of underweight associated with PTB may involve that deficiencies in micronutrients, decreased plasma volume, and increased risk of infection and inflammation, or other unidentified problems due to low BMI. Meanwhile, the possible biologic mechanism for the link between overweight/obesity and PTB is that overweight/obese women are at increased risk of metabolic disorders such as gestational diabetes mellitus and pregnancy-induced hypertension (24), which could lead to medically indicated PTB. Since rates of underweight and overweight/obesity among Chinese women were comparatively notable (25), overweight/obese women seemed at greater risk of PTB as well as their underweight counterparts, which revealed that women with reproductive age in China are facing a double problem considering PTB, attributable to maternal pre-conception BMI.

Hyperglycemia has been suggested to be associated with increased risk of pregnancy complications and adverse prenatal outcomes (11, 26). Billionnet et al. found that the risk of PTB was 5.8 and 3.1 times higher in type 1 diabetes and type 2 diabetes, separately, than in the no diabetes group (11). Baer et al. establised a predictive model for PTB using, 2339,696 California singleton livebirths from 2007 to 2012, and pre-existing diabetes was found to be the independent risk fator of PTB after adjusting mutilple confounders (27). Similar results were reported in Sweden (12), Australia (28), and among immigrant women (29). There was only one study directly focused on the association between maternal blood glucose levels before pregnancy and PTB, but they found null associations between all the elevated glucose groups and PTB when compared with reference group (FPG 1.0 ~ 4.4 mmol/L) (30). This discrepancy could possibly due to their small sample size (4,990 singleton births) which limited the statistical power to detect a significant association, or the differences in characteristics of study population and the definition of glucose groups.

Previously, maternal hypoglycemia during pregnancy has been linked to intra uterine growth retardation and low birth weight (31). Seldom study had precisely explored the effect of hypoglycemia on PTB. Ray et al. categorized 42,323 participants into quantiles according to 1-h glucose concentrations during 24–28 weeks' gestation, and reported a U-shaped relation between glucose and PTB plus small-for-gestational age, with highest adjusted OR at the hypoglycemia group (32). In the current study, we found maternal hypoglycemia before pregnancy was associated with an increased risk of PTB after we excluded participants who took hypoglycaemic agent at baseline. Women with hypoglycemia not caused by hypoglycaemic agent intake may have poor physical conditions, such as malnutrition and some other chronic diseases. Low maternal glucose might hinder growth-promoting aspects of the fetus' environment (31), then may result in pre-term delivery.

To date, very few studies have focused on the combined association of maternal BMI and FPG on adverse pregnancy outcomes. In a retrospective cohort study assessing the association between maternal pre-conception BMI, FPG during 24–28 weeks of gestation with risk of PTB among women with polycystic ovary syndrome, elevated FPG and obesity were jointly associated with a higher risk of PTB (33). However, BMI was categorized as <25, 25–30, and ≥30 kg/m^2^, so the combined effects of underweight and hypoglycemia on PTB was not explored in the study. Ricart et al. reported a greater risk of macrosomia, cesarean section, pregnancy-induced hypertension, and large for gestational age in the combined group of overweight and GDM than their single groups, but their effect on PTB was not significant (34). Our study indicated that combined abnormal pre-conception BMI and FPG has a greater risk of PTB, which highlights the importance of weight management in addition to glycemic control prior to conception.

The prevalence of overweight or obesity among reproductive-aged women is increasing worldwide (35), further exacerbating risk of adverse pregnancy outcomes in women with preexisting diabetes (36). The International Diabetes Federation estimated that nearly 21.3 million live births (16.2%) were affected by some form of hyperglycemia in pregnancy in 2017, and the vast majority of cases were in low- and middle-income countries (37). Meanwhile, both underweight and undernutrition remain a crucial health problem in low- and middle-income countries (38, 39). Our findings indicated that desirable pre-pregnancy weight and glucose monitoring is beneficial for reducing pre-term birth.

### Strengths and Limitations

The main strength of our study is its large sample size and nation-wide coverage with 2,907 counties/districts across 31 provinces and municipalities in Mainland China. This nation-wide universal pre-conception care in China provides a window to the future of the challenge of prenatal care in the next millennium, and we need to further evaluate its cost-effectiveness. However, there are some limitations should be mentioned. Firstly, lacking the information of maternal conditions during pregnancy, including maternal weight gain, hypoglycemic medication, and pregnancy-related complications of the current pregnancy, such as gestational hypertension or pre-eclampsia and gestational diabetes, so we could not adjust for these factors in the multivariable analysis, which could result in biased results. Further assessment of BMI during pregnancy or weight gain on PTB is needed. Secondly, detailed information regarding specific types of PTB were not collected, the association between the exposures and spontaneous PTB or iatrogenic PTB cannot be well-explored. Thirdly, this study was a population-based retrospective cohort study, there may be selection bias in the study population. In addition, although the participants were from both rural and urban China, most of the participants in our study were from rural areas (91.2%), which might limit the extrapolation of our results.

## Conclusions

In summary, we found that the associations between PTB and BMI varied with levels of FPG, and associations of PTB with FPG varied with levels of BMI. The risk of PTB was significantly associated with a synergistic effect due to abnormal weight and glycemia besides a conventional main effect derived from either of them in Chinese reproductive-aged women. Further attention for underweight and hypoglycemia women should not be ignored regarding the risk of PTB.

## Data Availability Statement

Data essential for the conclusion are included in this article. Additional data can be obtained from the corresponding author on a reasonable request.

## Ethics Statement

The current study was conducted according to the guidelines laid down in the Declaration of Helsinki, and all procedures involving humans were approved by the Institutional Research Review Board at the National Research Institute for Health and Family Planning. Written informed consent was obtained from all NFPCP participants. The patients/participants provided their written informed consent to participate in this study.

## Author Contributions

QX, QZ, YY, XM, and XL contributed to the conception and design of the work. QX contributed to the data analysis. QX and QZ contributed to the drafting the article. All authors contributed to the interpretation of the results, critical revision of the manuscript, and final approval of the manuscript.

## Conflict of Interest

The authors declare that the research was conducted in the absence of any commercial or financial relationships that could be construed as a potential conflict of interest.
