# Supplementary material for: Maternal Pre-conception Body Mass Index and Fasting Plasma Glucose With the Risk of Pre-term Birth: A Cohort Study Including 4.9 Million Chinese Women
Source: Front Reprod Health. 2021 Jun 15;3:622346. doi: 10.3389/frph.2021.622346 (PMC9580732; doi:10.3389/frph.2021.622346)
Supplement: Supplementary file 1 [file Table_1.docx]

**Number of Table: 4**

**Number of Figure: 5**

**Contents**

[Table S1. Comparison of baseline characteristics between included and excluded participants. 3](#_Toc70258821)

[Table S2. The combined associations of maternal preconception BMI and FPG with preterm birth, moderate preterm birth and very preterm birth using Chinese criterion of BMI. 5](#_Toc70258822)

[Table S3. The combined associations of maternal preconception BMI and FPG with preterm birth, moderate preterm birth and very preterm birth after excluding participants with self-reported diabetes and other chronic disease. 7](#_Toc70258823)

[Table S4. The combined associations of maternal preconception BMI and FPG with preterm birth according to delivery type. 9](#_Toc70258824)

[Figure S1. The combined associations of maternal preconception BMI and FPG with PTB, MPTB and VPTB. 11](#_Toc70258825)

[Figure S2. The exposure-response relationship of preconception BMI with and with MPTB, stratified by FPG. 12](#_Toc70258826)

[Figure S3. The exposure-response relationship of preconception BMI with and with VPTB, stratified by FPG. 13](#_Toc70258827)

[Figure S4. The exposure-response relationship of preconception FPG with and with MPTB, stratified by BMI. 14](#_Toc70258828)

[Figure S5. The exposure-response relationship of preconception FPG with and with VPTB, stratified by BMI 15](#_Toc70258829)

# Table S1. Comparison of baseline characteristics between included and excluded participants.

| **Characteristics** | **Included** | **Excluded** | **P value** |
| --- | --- | --- | --- |
| **N (%)** | 4987129 (97.6) | 123479 (2.4) |  |
| **BMI, kg/m^2^** |  |  | <0.001 |
| <18.5 | 675297 (13.5) | 13821 (11.2) |  |
| 18.5-24.9 | 3832440 (76.8) | 71264 (57.7) |  |
| 25.0-29.9 | 416706 (8.4) | 7963 (6.4) |  |
| ≥30.0 | 62686 (1.3) | 1324 (1.1) |  |
| NA | 0 (0.0) | 29107 (23.6) |  |
| **FPG, mmol/L** |  |  | <0.001 |
| <2.8 | 11088 (0.2) | 168 (0.1) |  |
| 2.8-5.5 | 4289316 (86.0) | 64543 (52.3) |  |
| 5.6-6.9 | 639661 (12.8) | 11104 (9.0) |  |
| ≥7.0 | 47064 (0.9) | 1325 (1.1) |  |
| NA | 0 (0.0) | 46339 (37.5) |  |
| **Age at last menstrual, years** |  |  | <0.001 |
| Mean±SD | 26.1±4.1 | 26.2±4.0 |  |
| 20-24 | 1994885 (40.0) | 44933 (36.4) |  |
| 25-29 | 2115194 (42.4) | 55867 (45.2) |  |
| 30-34 | 642345 (12.9) | 16948 (13.7) |  |
| 35-39 | 199390 (4.0) | 4955 (4.0) |  |
| 40-49 | 35315 (0.7) | 776 (0.6) |  |
| **Ethnicity** |  |  | 0.145 |
| Han | 4591716 (92.1) | 112843 (91.4) |  |
| Others | 338755 (6.8) | 8465 (6.9) |  |
| NA | 56658 (1.1) | 2171 (1.8) |  |
| **Education** |  |  | <0.001 |
| High school or above | 1808064 (36.3) | 53297 (43.2) |  |
| Low education | 3035338 (60.9) | 60944 (49.4) |  |
| NA | 143727 (2.9) | 9238 (7.5) |  |
| **Occupation** |  |  | <0.001 |
| Farmers | 3537200 (70.9) | 68803 (55.7) |  |
| Others | 1293287 (25.9) | 43509 (35.2) |  |
| NA | 156642 (3.1) | 11167 (9.0) |  |
| **Parity** |  |  | <0.001 |
| Nullipara | 3043053 (61.0) | 78413 (63.5) |  |
| Multipara | 1929055 (38.7) | 38178 (30.9) |  |
| NA | 15021 (0.3) | 6888 (5.6) |  |
| **Smoking** |  |  | <0.001 |
| No | 4899644 (98.2) | 105867 (85.7) |  |
| Yes | 24801 (0.5) | 646 (0.5) |  |
| NA | 62684 (1.3) | 16966 (13.7) |  |
| **Alcohol drinking** |  |  | <0.001 |
| No | 4767062 (95.6) | 101418 (82.1) |  |
| Yes | 151280 (3.0) | 4910 (4.0) |  |
| NA | 68787 (1.4) | 17151 (13.9) |  |
| **Adverse pregnancy history ^a^** |  |  | <0.001 |
| No | 4157294 (83.4) | 97112 (78.6) |  |
| Yes | 814810 (16.3) | 19479 (15.8) |  |
| NA | 15025 (0.3) | 6888 (5.6) |  |
| **Hypertension** |  |  | 0.0395 |
| No | 4905329 (98.4) | 114626 (92.8) |  |
| Yes | 74039 (1.5) | 1818 (1.5) |  |
| NA | 7761 (0.2) | 7035 (5.7) |  |
| **Thyroid dysfunction** |  |  | 0.019 |
| No | 4403374 (88.3) | 103456 (83.8) |  |
| Yes | 576630 (11.6) | 13254 (10.7) |  |
| NA | 7125 (0.1) | 6769 (5.5) |  |
| **Anemia** |  |  | <0.001 |
| No | 4646641 (93.2) | 106124 (85.9) |  |
| Yes | 328191 (6.6) | 6456 (5.2) |  |
| NA | 12297 (0.2) | 10899 (8.8) |  |
| **Infectious disease** |  |  | <0.001 |
| No | 4502465 (90.3) | 75848 (61.4) |  |
| Yes | 265087 (5.3) | 6365 (5.2) |  |
| NA | 219577 (4.4) | 41266 (33.4) |  |
| **Neonatal sex** |  |  | <0.001 |
| Male | 2591998 (52.0) | 66602 (53.9) |  |
| Female | 2389131 (47.9) | 56544 (45.8) |  |
| NA | 6000 (0.1) | 333 (0.3) |  |
| **Caesarean** |  |  | <0.001 |
| No | 3553810 (71.3) | 72152 (58.4) |  |
| Yes | 1433319 (28.7) | 51278 (41.5) |  |
| NA | 0 (0.0) | 49 (0.0) |  |
| **Habitant** |  |  | <0.001 |
| Rural | 4549991 (91.2) | 104766 (84.8) |  |
| Urban | 436711 (8.8) | 18697 (15.1) |  |
| NA | 427 (0.0) | 16 (0.0) |  |

^a^ Adverse pregnancy history included history of spontaneous abortion, stillbirth, preterm birth, or induced abortion.

BMI, body mass index; FPG, fasting plasma glucose.

# Table S2. The combined associations of maternal preconception BMI and FPG with preterm birth, moderate preterm birth and very preterm birth using Chinese criterion of BMI.

| **Classification** |  | **Preterm birth** | | |  | **Moderate preterm birth** | | |  | **Very preterm birth** | | |
| --- | --- | --- | --- | --- | --- | --- | --- | --- | --- | --- | --- | --- |
|  |  | **n (%)** | **Crude OR (95% CI)** | **Multivariable adjusted OR (95% CI) ^#^** |  | **n (%)** | **Crude OR (95% CI)** | **Multivariable adjusted OR (95% CI) ^#^** |  | **n (%)** | **Crude OR (95% CI)** | **Multivariable adjusted OR (95% CI) ^#^** |
| Hypoglycemia | Underweight | 140 (8.0) | 1.23 (1.03-1.46) | 1.25 (1.05-1.48) |  | 95 (5.6) | 1.18 (0.96-1.45) | 1.19 (0.96-1.46) |  | 45 (2.7) | 1.35 (1.01-1.82) | 1.4 (1.04-1.88) |
|  | Normal weight | 621 (7.7) | 1.19 (1.09-1.29) | 1.16 (1.07-1.26) |  | 428 (5.5) | 1.15 (1.05-1.27) | 1.13 (1.03-1.25) |  | 193 (2.5) | 1.27 (1.1-1.46) | 1.22 (1.06-1.41) |
|  | Overweight | 75 (6.9) | 1.04 (0.82-1.32) | 0.96 (0.76-1.22) |  | 51 (4.8) | 1 (0.75-1.32) | 0.93 (0.7-1.24) |  | 24 (2.3) | 1.14 (0.76-1.71) | 1.03 (0.69-1.54) |
|  | Obesity | 21 (9.6) | 1.5 (0.96-2.35) | 1.39 (0.88-2.18) |  | 13 (6.2) | 1.31 (0.75-2.3) | 1.22 (0.7-2.14) |  | 8 (3.9) | 1.96 (0.97-3.97) | 1.79 (0.88-3.63) |
| Normal glycemia | Underweight | 41374 (7.0) | 1.07 (1.05-1.08) | 1.09 (1.08-1.11) |  | 29862 (5.2) | 1.09 (1.07-1.1) | 1.11 (1.09-1.12) |  | 11512 (2.1) | 1.02 (1-1.04) | 1.06 (1.04-1.08) |
|  | Normal weight | 203649 (6.6) | 1 | 1 |  | 144291 (4.8) | 1 | 1 |  | 59358 (2) | 1 (1-1) | 1 (1-1) |
|  | Overweight | 36012 (7.1) | 1.09 (1.07-1.1) | 1.04 (1.03-1.05) |  | 25723 (5.2) | 1.09 (1.08-1.11) | 1.05 (1.04-1.07) |  | 10289 (2.1) | 1.06 (1.04-1.09) | 1 (0.98-1.03) |
|  | Obesity | 8358 (7.6) | 1.16 (1.14-1.19) | 1.09 (1.06-1.11) |  | 5938 (5.5) | 1.16 (1.13-1.2) | 1.1 (1.07-1.13) |  | 2420 (2.3) | 1.15 (1.11-1.2) | 1.06 (1.02-1.11) |
| Prediabetes | Underweight | 5444 (7.1) | 1.08 (1.05-1.11) | 1.09 (1.06-1.13) |  | 3899 (5.2) | 1.09 (1.05-1.12) | 1.1 (1.07-1.14) |  | 1545 (2.1) | 1.05 (1-1.1) | 1.08 (1.03-1.14) |
|  | Normal weight | 30471 (6.9) | 1.05 (1.04-1.07) | 1.04 (1.03-1.05) |  | 21853 (5.1) | 1.06 (1.05-1.08) | 1.05 (1.04-1.07) |  | 8618 (2.1) | 1.02 (1-1.04) | 1.01 (0.99-1.03) |
|  | Overweight | 7403 (7.7) | 1.18 (1.15-1.21) | 1.12 (1.09-1.14) |  | 5265 (5.6) | 1.19 (1.15-1.22) | 1.12 (1.09-1.16) |  | 2138 (2.4) | 1.17 (1.12-1.22) | 1.1 (1.05-1.15) |
|  | Obesity | 2302 (8.7) | 1.35 (1.29-1.41) | 1.24 (1.19-1.3) |  | 1642 (6.4) | 1.36 (1.29-1.43) | 1.26 (1.2-1.32) |  | 660 (2.7) | 1.33 (1.23-1.43) | 1.21 (1.12-1.31) |
| Diabetes | Underweight | 447 (7.6) | 1.16 (1.05-1.27) | 1.18 (1.07-1.3) |  | 313 (5.4) | 1.14 (1.02-1.28) | 1.16 (1.03-1.3) |  | 134 (2.4) | 1.19 (1-1.41) | 1.23 (1.04-1.46) |
|  | Normal weight | 2228 (7.7) | 1.17 (1.12-1.23) | 1.16 (1.11-1.21) |  | 1562 (5.5) | 1.16 (1.1-1.22) | 1.14 (1.09-1.2) |  | 666 (2.4) | 1.2 (1.11-1.3) | 1.19 (1.1-1.28) |
|  | Overweight | 713 (8.6) | 1.33 (1.24-1.44) | 1.25 (1.16-1.35) |  | 513 (6.4) | 1.35 (1.24-1.48) | 1.27 (1.16-1.39) |  | 200 (2.6) | 1.28 (1.12-1.48) | 1.19 (1.04-1.38) |
|  | Obesity | 404 (10.6) | 1.68 (1.51-1.86) | 1.52 (1.37-1.69) |  | 291 (7.9) | 1.71 (1.51-1.92) | 1.55 (1.38-1.75) |  | 113 (3.2) | 1.61 (1.34-1.94) | 1.46 (1.21-1.76) |

BMI, body mass index. Underweight (BMI <18.5 kg/m^2^); normal weight (BMI 18.5-23.9 kg/m^2^); overweight (BMI 24.0-27.9 kg/m^2^); obesity (BMI ≥28.0 kg/m^2^);

FPG, fasting plasma glucose. Hypoglycemia (FPG ≤2.7 mmol/L); normal glucose (FPG 2.8-5.5 mmol/L); prediabetes (FPG 5.6–6.9 mmol/L); diabetes (FPG ≥7.0 mmol/L).

Moderate preterm birth (34 to <37 weeks), Very preterm birth (28 to <34 weeks); OR, odd ratio. Multivariable-adjusted OR (95% CI) were adjusted for characteristics of women (age, education, ethnic, occupation), parity, smoking and alcohol drinking before or during early pregnancy, history of adverse pregnancy outcomes, history of hypertension, thyroid dysfunction, anemia, infectious disease, and sex of child.

# Table S3. The combined associations of maternal preconception BMI and FPG with preterm birth, moderate preterm birth and very preterm birth after excluding participants with self-reported diabetes and other chronic diseases.

| **Classification** |  | **Preterm birth** | | |  | **Moderate preterm birth** | | |  | **Very preterm birth** | | |
| --- | --- | --- | --- | --- | --- | --- | --- | --- | --- | --- | --- | --- |
|  |  | **n (%)** | **Crude OR (95% CI)** | **Multivariable adjusted OR (95% CI) ^#^** |  | **n (%)** | **Crude OR (95% CI)** | **Multivariable adjusted OR (95% CI) ^#^** |  | **n (%)** | **Crude OR (95% CI)** | **Multivariable adjusted OR (95% CI) ^#^** |
| Hypoglycemia | Underweight | 140 (8.0) | 1.23 (1.03-1.46) | 1.20 (1.00-1.44) |  | 95 (5.6) | 1.17 (0.95-1.44) | 1.12 (0.89-1.40) |  | 45 (2.7) | 1.35 (1.01-1.82) | 1.40 (1.03-1.91) |
|  | Normal weight | 663 (7.8) | 1.19 (1.10-1.29) | 1.10 (1.01-1.20) |  | 455 (5.5) | 1.15 (1.05-1.27) | 1.10 (0.99-1.21) |  | 208 (2.6) | 1.28 (1.12-1.47) | 1.11 (0.96-1.30) |
|  | Overweight | 44 (6.1) | 0.92 (0.68-1.25) | 0.85 (0.62-1.17) |  | 32 (4.5) | 0.95 (0.66-1.35) | 0.89 (0.62-1.28) |  | 12 (1.8) | 0.86 (0.49-1.53) | 0.77 (0.42-1.39) |
|  | Obesity | 10 (11.8) | 1.88 (0.97-3.64) | 1.65 (0.83-3.31) |  | 5 (6.3) | 1.33 (0.54-3.28) | 1.30 (0.52-3.22) |  | 5 (6.3) | 3.23 (1.30-7.97) | 2.48 (0.90-6.80) |
| Normal glucose | Underweight | 41282 (7.0) | 1.06 (1.05-1.07) | 1.10 (1.08-1.11) |  | 29791 (5.2) | 1.08 (1.07-1.09) | 1.11 (1.09-1.12) |  | 11491 (2.1) | 1.01 (0.99-1.03) | 1.07 (1.05-1.09) |
|  | Normal weight | 219066 (6.6) | 1 | 1 |  | 155239 (4.8) | 1 | 1 |  | 63827 (2.0) | 1 | 1 |
|  | Overweight | 24761 (7.3) | 1.12 (1.10-1.13) | 1.06 (1.05-1.08) |  | 17775 (5.4) | 1.13 (1.11-1.15) | 1.08 (1.06-1.10) |  | 6986 (2.2) | 1.08 (1.05-1.11) | 1.01 (0.99-1.04) |
|  | Obesity | 3678 (7.6) | 1.16 (1.12-1.20) | 1.08 (1.04-1.12) |  | 2571 (5.4) | 1.14 (1.1-1.19) | 1.07 (1.03-1.12) |  | 1107 (2.4) | 1.19 (1.12-1.27) | 1.10 (1.03-1.17) |
| Prediabetes | Underweight | 5428 (7.1) | 1.07 (1.04-1.10) | 1.10 (1.07-1.13) |  | 3886 (5.2) | 1.08 (1.05-1.12) | 1.10 (1.06-1.14) |  | 1542 (2.1) | 1.04 (0.99-1.10) | 1.09 (1.04-1.15) |
|  | Normal weight | 33267 (6.9) | 1.05 (1.04-1.06) | 1.04 (1.03-1.05) |  | 23836 (5.1) | 1.06 (1.05-1.08) | 1.05 (1.03-1.06) |  | 9431 (2.1) | 1.02 (1.00-1.05) | 1.01 (0.99-1.03) |
|  | Overweight | 5782 (8.3) | 1.27 (1.24-1.31) | 1.19 (1.15-1.22) |  | 4113 (6.0) | 1.27 (1.23-1.32) | 1.19 (1.15-1.23) |  | 1669 (2.5) | 1.26 (1.20-1.32) | 1.18 (1.12-1.24) |
|  | Obesity | 1019 (8.6) | 1.32 (1.24-1.41) | 1.21 (1.13-1.30) |  | 728 (6.3) | 1.33 (1.23-1.43) | 1.23 (1.14-1.33) |  | 291 (2.6) | 1.29 (1.15-1.45) | 1.18 (1.04-1.33) |
| Diabetes | Underweight | 442 (7.5) | 1.14 (1.04-1.26) | 1.17 (1.05-1.29) |  | 309 (5.4) | 1.13 (1.01-1.26) | 1.13 (1.00-1.28) |  | 133 (2.4) | 1.18 (0.99-1.40) | 1.26 (1.05-1.50) |
|  | Normal weight | 2480 (7.8) | 1.18 (1.14-1.23) | 1.18 (1.13-1.23) |  | 1742 (5.6) | 1.17 (1.12-1.23) | 1.16 (1.10-1.22) |  | 738 (2.4) | 1.21 (1.12-1.30) | 1.23 (1.14-1.32) |
|  | Overweight | 639 (9.2) | 1.42 (1.31-1.54) | 1.31 (1.20-1.42) |  | 463 (6.8) | 1.45 (1.32-1.6) | 1.33 (1.21-1.47) |  | 176 (2.7) | 1.34 (1.16-1.56) | 1.26 (1.08-1.47) |
|  | Obesity | 201 (10.7) | 1.69 (1.46-1.96) | 1.58 (1.36-1.84) |  | 147 (8.1) | 1.75 (1.48-2.07) | 1.64 (1.38-1.95) |  | 54 (3.1) | 1.56 (1.19-2.05) | 1.45 (1.10-1.92) |

BMI, body mass index. Underweight (BMI <18.5 kg/m^2^); normal weight (BMI 18.5-23.9 kg/m^2^); overweight (BMI 24.0-27.9 kg/m^2^); obesity (BMI ≥28.0 kg/m^2^);

FPG, fasting plasma glucose. Hypoglycemia (FPG ≤2.7 mmol/L); normal glucose (FPG 2.8-5.5 mmol/L); prediabetes (FPG 5.6–6.9 mmol/L); diabetes (FPG ≥7.0 mmol/L).

Moderate preterm birth (34 to <37 weeks), Very preterm birth (28 to <34 weeks); OR, odd ratio. Multivariable-adjusted OR (95% CI) were adjusted for characteristics of women (age, education, ethnic, occupation), parity, smoking and alcohol drinking before or during early pregnancy, history of adverse pregnancy outcomes, history of hypertension, thyroid dysfunction, anemia, infectious disease, and sex of child.

# Table S4. The combined associations of maternal preconception BMI and FPG with preterm birth according to delivery type.

| **Classification** |  | **Cesarean (Yes)** | | |  | **Cesarean (No)** | | |
| --- | --- | --- | --- | --- | --- | --- | --- | --- |
|  |  | **n (%)** | **Crude OR (95% CI)** | **Multivariable adjusted OR (95% CI) ^#^** |  | **n (%)** | **Crude OR (95% CI)** | **Multivariable adjusted OR (95% CI) ^#^** |
| Hypoglycemia | Underweight | 27 (6.4) | 0.90 (0.61-1.33) | 0.96 (0.65-1.42) |  | 113 (8.5) | 1.34 (1.11-1.63) | 1.34 (1.11-1.63) |
|  | Normal weight | 167 (7.9) | 1.14 (0.98-1.34) | 1.15 (0.98-1.35) |  | 496 (7.7) | 1.21 (1.10-1.32) | 1.16 (1.06-1.27) |
|  | Overweight | 12 (4.6) | 0.64 (0.36-1.14) | 0.59 (0.33-1.05) |  | 32 (7.0) | 1.08 (0.75-1.55) | 0.99 (0.69-1.42) |
|  | Obesity | 5 (15.6) | 2.46 (0.95-6.38) | 2.21 (0.85-5.78) |  | 5 (9.4) | 1.50 (0.60-3.78) | 1.46 (0.58-3.67) |
| Normal glucose | Underweight | 10157 (7.0) | 1.00 (0.97-1.02) | 1.05 (1.03-1.07) |  | 31217 (7.0) | 1.09 (1.08-1.10) | 1.11 (1.09-1.12) |
|  | Normal weight | 64830 (7.0) | 1 | 1 |  | 154650 (6.5) | 1 | 1 |
|  | Overweight | 10425 (8.0) | 1.15 (1.13-1.18) | 1.08 (1.05-1.10) |  | 14415 (6.9) | 1.07 (1.05-1.09) | 1.03 (1.01-1.05) |
|  | Obesity | 1771 (8.5) | 1.23 (1.17-1.30) | 1.12 (1.07-1.18) |  | 1928 (6.9) | 1.07 (1.02-1.12) | 1.02 (0.97-1.07) |
| Prediabetes | Underweight | 1348 (7.1) | 1.01 (0.96-1.07) | 1.05 (1.00-1.12) |  | 4096 (7.1) | 1.10 (1.06-1.13) | 1.11 (1.07-1.15) |
|  | Normal weight | 10469 (7.5) | 1.08 (1.05-1.10) | 1.06 (1.03-1.08) |  | 22876 (6.7) | 1.04 (1.02-1.05) | 1.03 (1.02-1.05) |
|  | Overweight | 2671 (9.2) | 1.34 (1.28-1.39) | 1.22 (1.17-1.27) |  | 3133 (7.6) | 1.19 (1.15-1.24) | 1.14 (1.10-1.18) |
|  | Obesity | 542 (9.4) | 1.38 (1.26-1.51) | 1.22 (1.12-1.34) |  | 485 (7.8) | 1.22 (1.11-1.34) | 1.15 (1.05-1.26) |
| Diabetes | Underweight | 106 (7.7) | 1.10 (0.91-1.35) | 1.16 (0.95-1.42) |  | 341 (7.5) | 1.17 (1.05-1.31) | 1.19 (1.06-1.32) |
|  | Normal weight | 816 (8.7) | 1.27 (1.18-1.36) | 1.25 (1.16-1.34) |  | 1679 (7.4) | 1.15 (1.09-1.21) | 1.13 (1.07-1.19) |
|  | Overweight | 315 (10.5) | 1.55 (1.38-1.74) | 1.41 (1.25-1.58) |  | 330 (8.2) | 1.28 (1.15-1.44) | 1.21 (1.08-1.36) |
|  | Obesity | 106 (11.3) | 1.69 (1.38-2.07) | 1.47 (1.20-1.80) |  | 99 (10.2) | 1.64 (1.33-2.01) | 1.54 (1.25-1.89) |

BMI, body mass index; FPG, fasting plasma glucose; OR, odd ratio. Multivariable-adjusted OR (95% CI) were adjusted for characteristics of women (age, education, ethnic, occupation), parity, smoking and alcohol drinking before or during early pregnancy, history of adverse pregnancy outcomes, history of hypertension, thyroid dysfunction, anemia, infectious disease and sex of child.


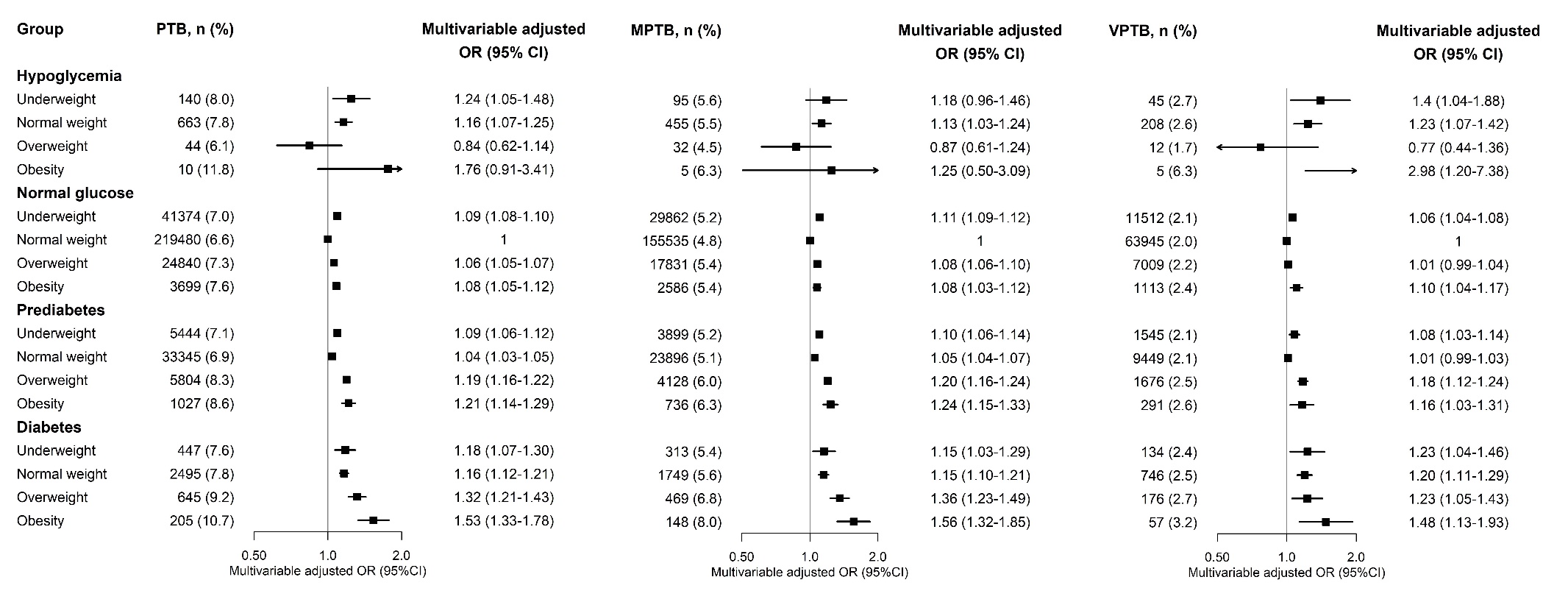


# Figure S1. The combined associations of maternal preconception BMI and FPG with PTB, MPTB and VPTB.

BMI, body mass index; FPG, fasting plasma glucose; PTB, preterm birth (28 to <37 weeks); MPTB, moderate preterm birth (34 to <37 weeks); VPTB, very preterm birth (28 to <34 weeks); OR, odd ratio. Multivariable-adjusted OR (95% CI) were adjusted for characteristics of women (age, education, ethnic, occupation), parity, smoking and alcohol drinking before or during early pregnancy, history of adverse pregnancy outcomes, history of hypertension, thyroid dysfunction, anemia, infectious disease, and sex of child.


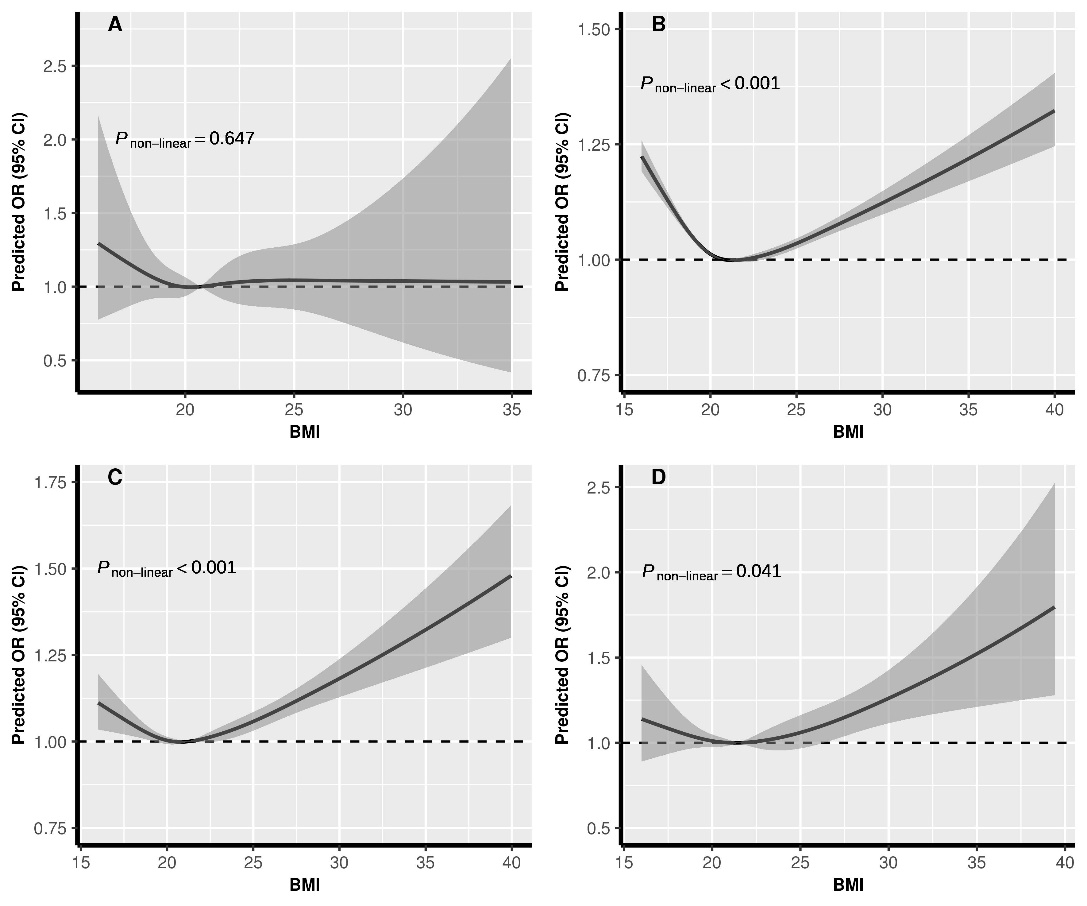


# Figure S2. The exposure-response relationship of preconception BMI with and with MPTB, stratified by FPG.

BMI, body mass index; FPG, fasting plasma glucose; MPTB (moderate PTB, 34 to <37 weeks). A: Hypoglycemia; B: Normal glucose; C: Prediabetes; D: Diabetes. In the graph, black lines and shaded grey areas represent predicted ORs and 95% CIs, respectively. The models were adjusted for characteristics of women (age, education, ethnic, occupation), parity, smoking and alcohol drinking before or during early pregnancy, history of adverse pregnancy outcomes, history of hypertension, thyroid dysfunction, anemia, infectious disease, and sex of child.


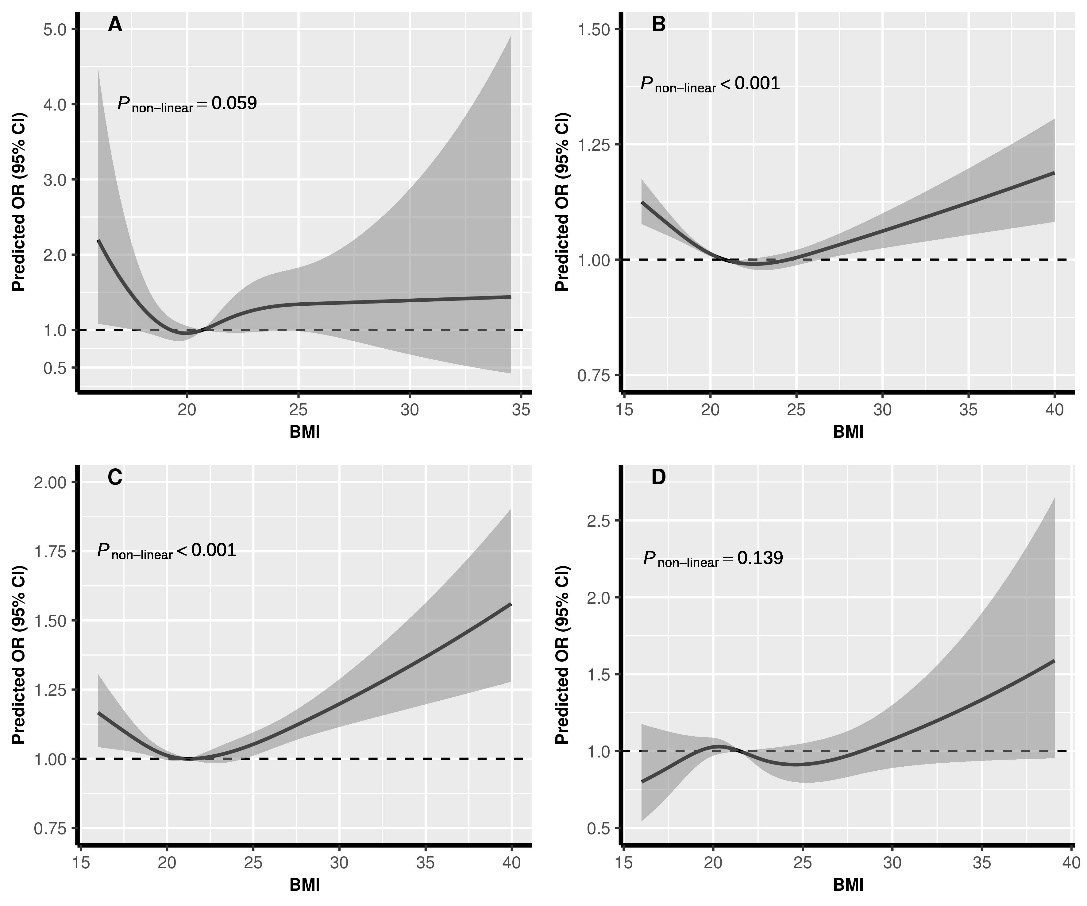


# Figure S3. The exposure-response relationship of preconception BMI with and with VPTB, stratified by FPG.

BMI, body mass index; FPG, fasting plasma glucose; VPTB (very PTB, 28 to <34 weeks). A: Hypoglycemia; B: Normal glucose; C: Prediabetes; D: Diabetes. In the graph, black lines and shaded grey areas represent predicted ORs and 95% CIs, respectively. The models were adjusted for characteristics of women (age, education, ethnic, occupation), parity, smoking and alcohol drinking before or during early pregnancy, history of adverse pregnancy outcomes, history of hypertension, thyroid dysfunction, anemia, infectious disease, and sex of child.


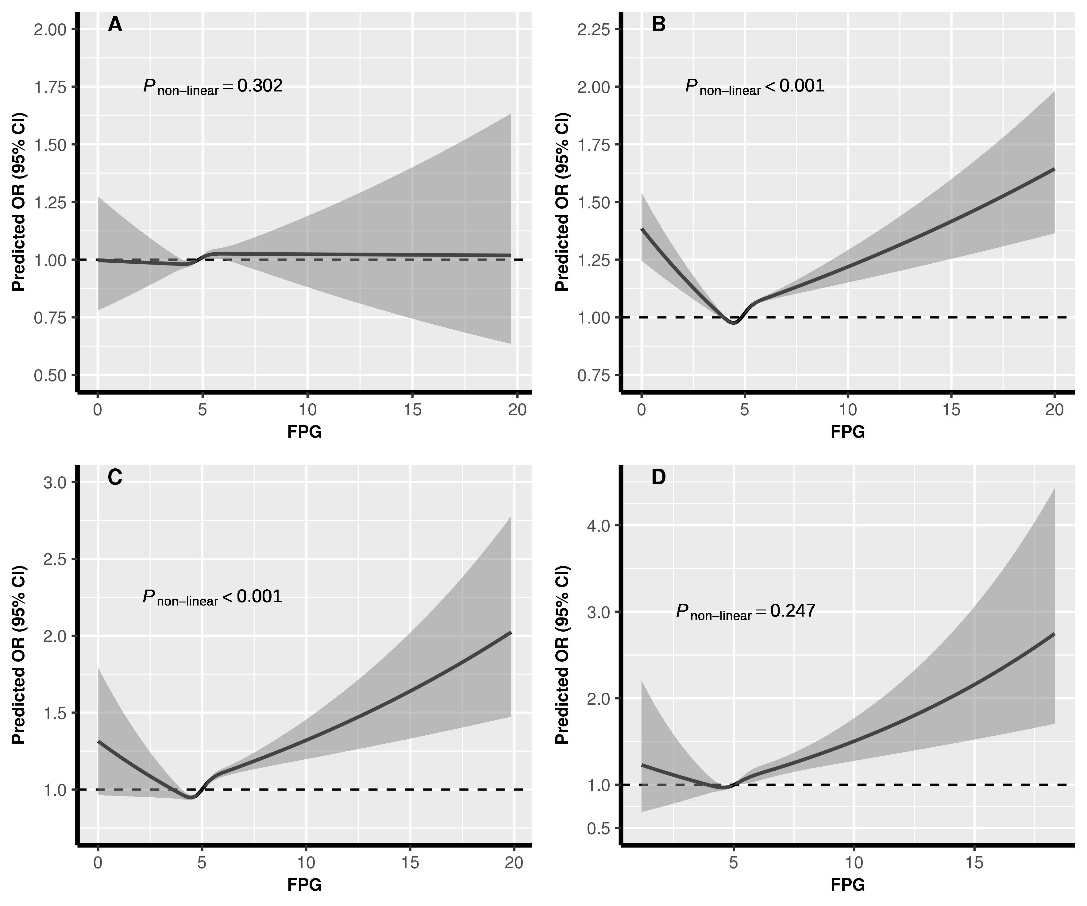


# Figure S4. The exposure-response relationship of preconception FPG with and with MPTB, stratified by BMI.

BMI, body mass index; FPG, fasting plasma glucose; MPTB (moderate PTB, 34 to <37 weeks). A: Underweight; B: Normal weight; C: Overweight; D: Obesity. In the graph, black lines and shaded grey areas represent predicted ORs and 95% CIs, respectively. The models were adjusted for characteristics of women (age, education, ethnic, occupation), parity, smoking and alcohol drinking before or during early pregnancy, history of adverse pregnancy outcomes, history of hypertension, thyroid dysfunction, anemia, infectious disease, and sex of child.


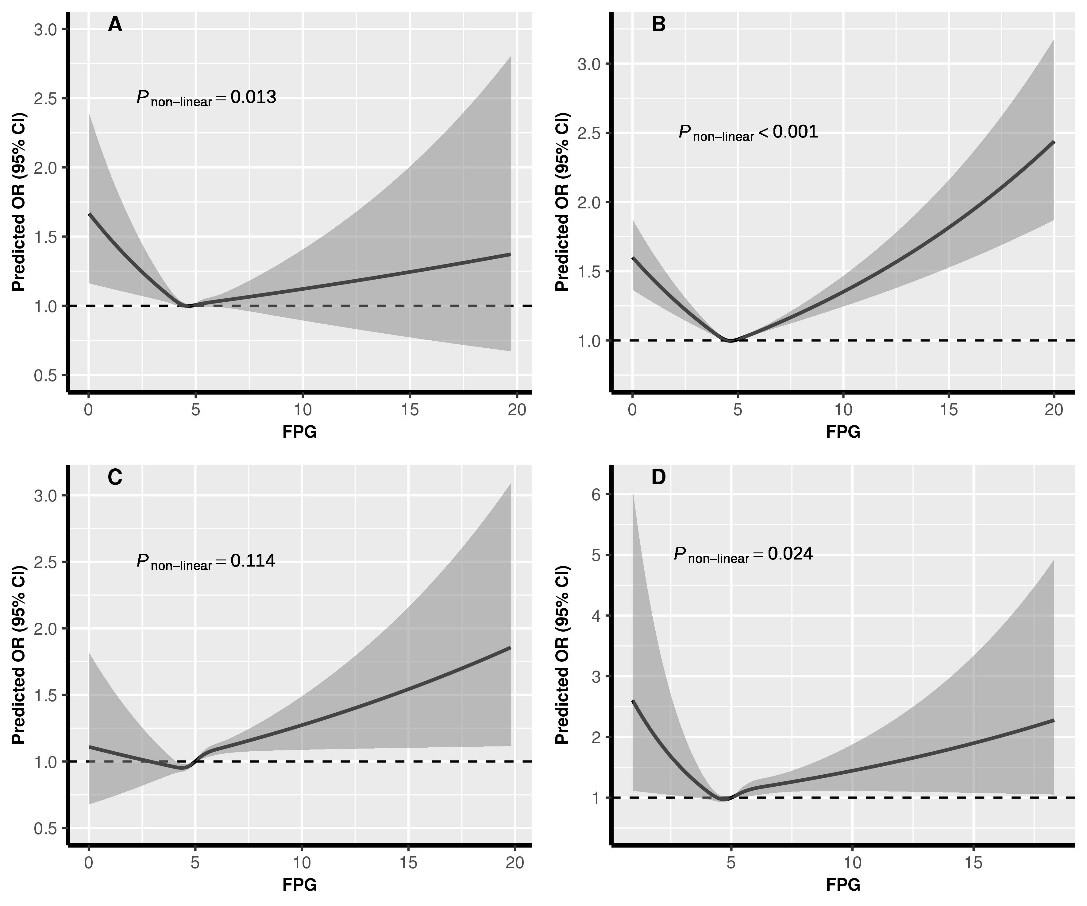


# Figure S5. The exposure-response relationship of preconception FPG with and with VPTB, stratified by BMI

BMI, body mass index; FPG, fasting plasma glucose; VPTB (very PTB, 28 to <34 weeks). A: Underweight; B: Normal weight; C: Overweight; D: Obesity. In the graph, black lines and shaded grey areas represent predicted ORs and 95% CIs, respectively. The models were adjusted for characteristics of women (age, education, ethnic, occupation), parity, smoking and alcohol drinking before or during early pregnancy, history of adverse pregnancy outcomes, history of hypertension, thyroid dysfunction, anemia, infectious disease, and sex of child.
